# Structural basis for the antagonistic roles of RNP-8 and GLD-3 in GLD-2 poly(A)-polymerase activity

**DOI:** 10.1261/rna.056598.116

**Published:** 2016-08

**Authors:** Katharina Nakel, Fabien Bonneau, Claire Basquin, Bianca Habermann, Christian R. Eckmann, Elena Conti

**Affiliations:** 1 Department of Structural Cell Biology, Max-Planck-Institute of Biochemistry, D-82152 Martinsried, Germany; 2 Department of Genetics, Martin-Luther-University of Halle-Wittenberg, Institute of Biology, 06120 Halle (Saale), Germany

**Keywords:** translational regulation, cytoplasmic polyadenylation, nucleotidyl-transferase, germline development, *C. elegans*

## Abstract

Cytoplasmic polyadenylation drives the translational activation of specific mRNAs in early metazoan development and is performed by distinct complexes that share the same catalytic poly(A)-polymerase subunit, GLD-2. The activity and specificity of GLD-2 depend on its binding partners. In *Caenorhabditis elegans*, GLD-2 promotes spermatogenesis when bound to GLD-3 and oogenesis when bound to RNP-8. GLD-3 and RNP-8 antagonize each other and compete for GLD-2 binding. Following up on our previous mechanistic studies of GLD-2–GLD-3, we report here the 2.5 Å resolution structure and biochemical characterization of a GLD-2–RNP-8 core complex. In the structure, RNP-8 embraces the poly(A)-polymerase, docking onto several conserved hydrophobic hotspots present on the GLD-2 surface. RNP-8 stabilizes GLD-2 and indirectly stimulates polyadenylation. RNP-8 has a different amino-acid sequence and structure as compared to GLD-3. Yet, it binds the same surfaces of GLD-2 by forming alternative interactions, rationalizing the remarkable versatility of GLD-2 complexes.

## INTRODUCTION

The poly(A) tail is a key post-transcriptional modification that impacts on the stability, export and translational efficiency of the vast majority of eukaryotic mRNAs (for review, see Moore and Proudfoot 2009 and Eckmann et al. 2011). The shortening of the poly(A) tail by cytoplasmic deadenylases is linked to translational repression and generally to the decay of the deadenylated mRNA. In germ cells and neuronal synapses in particular, deadenylation also serves to store the transcripts in a dormant state until translation is resumed upon the extension of the poly(A) tail by cytoplasmic poly(A)-polymerases (for review, see Weill et al. 2012 and Norbury 2013).

GLD-2 (germline development defective 2) is a highly conserved cytoplasmic poly(A)-polymerase in metazoans. It was discovered in *Caenorhabditis elegans* for its role in meiotic entry (Wang et al. 2002) and has since been identified and studied in other species, including *X. laevis*, *D. melanogaster*, and *M. musculus* (Barnard et al. 2004; Kwak et al. 2004; Rouhana and Wickens 2007; Benoit et al. 2008; Sartain et al. 2011). GLD-2 orthologs control many aspects of germline development, including the production of male and female gametes (Kadyk and Kimble 1998; Eckmann et al. 2004; Hansen et al. 2004; Kim et al. 2009, 2010; Sartain et al. 2011; Cui et al. 2013; Nousch et al. 2014). In addition, they are involved in the formation of long-term memory in the brain (Kwak et al. 2008; Udagawa et al. 2012).

GLD-2 is a member of the noncanonical family of poly(A)-polymerases (Martin and Keller 2007; Schmidt and Norbury 2010). It lacks the RNA recognition motif (RRM) fold characteristic of the canonical nuclear poly(A)-polymerase and associates with a variety of binding partners that in general activate and target it to specific transcripts (Wang et al. 2002; Barnard et al. 2004; Eckmann et al. 2004; Suh et al. 2006; Papin et al. 2008; Kim et al. 2009, 2010; Cragle and MacNicol 2014). *Caenorhabditis elegans* GLD-3 is arguably the best studied GLD-2–binding partner (Eckmann et al. 2002). GLD-3 contains four noncanonical KH domains (Nakel et al. 2010). It also contains an N-terminal region that wraps around GLD-2 and increases the RNA-binding properties and the stability of the poly(A)-polymerase, thereby promoting polyadenylation (Nakel et al. 2015).

Functionally, the GLD-2–GLD-3 complex controls the transition from mitosis to meiosis (Wang et al. 2002; Crittenden et al. 2003; Eckmann et al. 2004) and spermatogenesis (Eckmann et al. 2002, 2004; Kim et al. 2009). While GLD-3 favors sperm fate, another GLD-2 binding protein, RNP-8, favors oocyte fate (Kim et al. 2009, 2010). RNP-8 and GLD-3 genetically antagonize each other for gamete production and form separate complexes with GLD-2 in vitro and in vivo (Kim et al. 2009, 2010). However, RNP-8 does not share apparent sequence similarity with GLD-3, raising the question of how the two proteins can compete for GLD-2 binding and activation. In this work, we used biochemical and structural studies to obtain insights into the molecular mechanisms.

## RESULTS AND DISCUSSION

**FIGURE 1.**
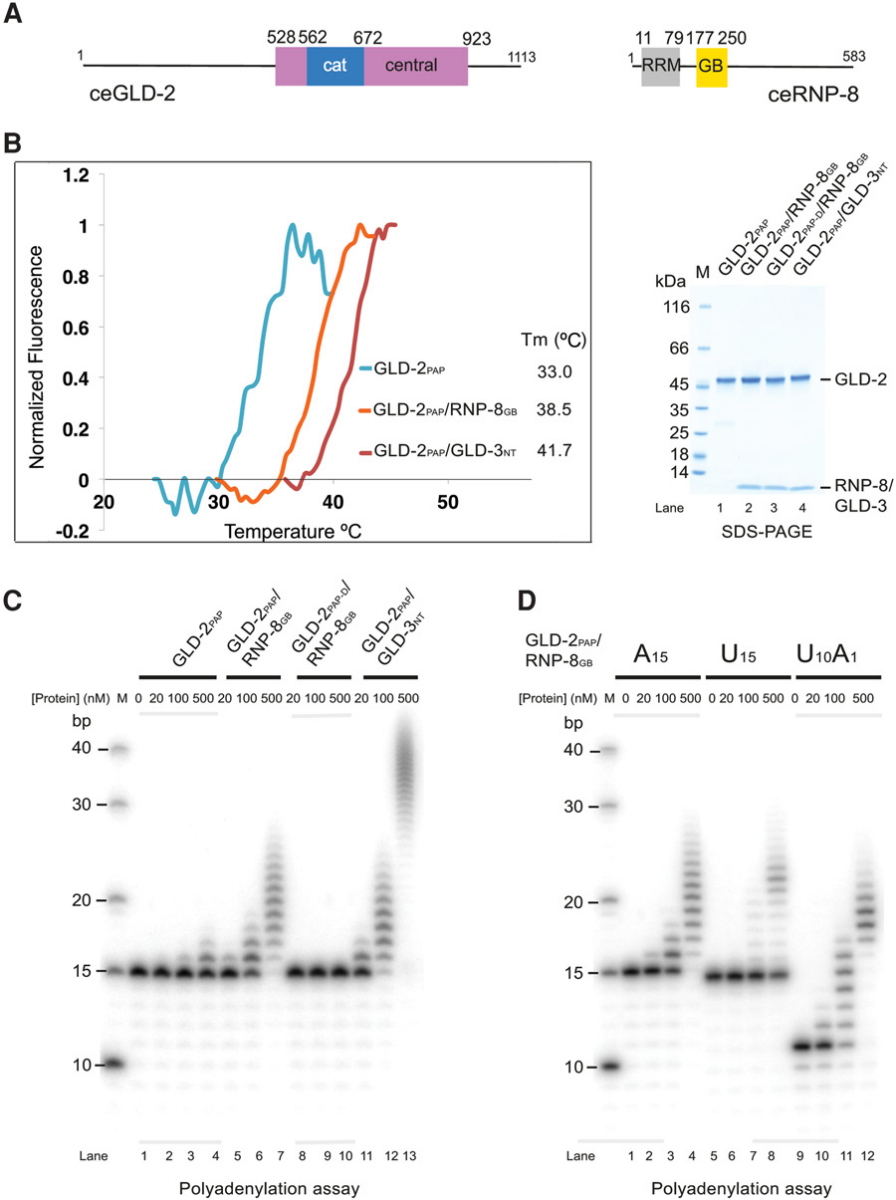
Activity of a minimal GLD-2–RNP-8 complex. (*A*) Schematic representation of the domain structure of *C. elegans* GLD-2 and RNP-8. Globular domains are shown as rectangles and low-complexity sequences as lines. The catalytic (cat) and central domains of GLD-2 are colored in blue and pink, respectively. As in other nucleotidyltransferases, the central domain is composed of two noncontiguous polypeptide segments, which correspond in the structure to helix α1 and helices α4–α8 for the segments preceding and following the catalytic domain, respectively (see also Fig. 2). The RNA recognition motif (RRM) and the GLD-2-binding (GB) domains of RNP-8 are colored in gray and yellow, respectively. (*B*) Protein stability of GLD-2_PAP_–RNP-8_GB_, GLD-2_PAP_–GLD-3_NT_, and GLD-2_PAP_ as determined by thermofluor experiments. The normalized curves and the corresponding melting temperatures are shown on the *left*. The Coomassie-stained 4%–15% Bio-Rad TGX SDS-PAGE gel of the proteins used in the thermofluor and poly(A) polymerase assays (*C*,*D*) are shown on the *right* and in Supplemental Figure 1C. (*C*) Polyadenylation assay of *C. elegans* GLD-2_PAP_ or GLD-2_PAP-D_ in complex with either RNP-8_GB_ or GLD-3_NT_ (Nakel et al. 2015). (*D*) Polyadenylation assay of GLD-2_PAP_–RNP-8_GB_ (0, 20, 100, and 500 nM) in the presence of 5′-^32^P-labeled A_15_, U_15_, or U_10_A_1_ oligomers (100 nM).

We previously characterized a nucleotidyl-transferase region of *C. elegans* GLD-2 suitable for biochemical and structural studies with the corresponding binding domain of GLD-3 (Nakel et al. 2015). We will refer to these fragments as GLD-2_PAP_ for the wild-type enzyme, and GLD-2_PAP-D_ for the catalytically dead D668A mutant (Fig. 1A; Materials and Methods). For GLD-3, we will refer to the N-terminal GLD-2 binding domain as GLD-3_NT_ (residues 13–88). Yeast-two-hybrid assays have identified a 39-residue segment in the unstructured C-terminal region of RNP-8 (residues 186–224) as the GLD-2 interaction site (Kim et al. 2009). We coexpressed GLD-2_PAP_ with a slightly larger RNP-8 fragment (residues 171–250), and found it proteolyzed spontaneously to residues 177–250 (referred to as RNP-8_GB_ for GLD-2 binding) (Fig. 1A; Supplemental Fig. 1A,B). GLD-2_PAP_ and RNP-8_GB_ co-purified in a binary complex with increased stability as compared to GLD-2_PAP_ in isolation (Fig. 1B; Supplemental Fig. 1C). GLD-2_PAP_–RNP-8_GB_ had poly(A)-polymerase activity in vitro on an A_15_ RNA substrate, albeit less robust than for GLD-2_PAP_–GLD-3_NT_ (Fig. 1C). Finally, GLD-2_PAP_–RNP-8_GB_ showed a preference for an RNA substrate with adenosines at the 3′ end (Fig. 1D), similarly to GLD-2_PAP_–GLD-3_NT_ (Nakel et al. 2015).

**FIGURE 2.**
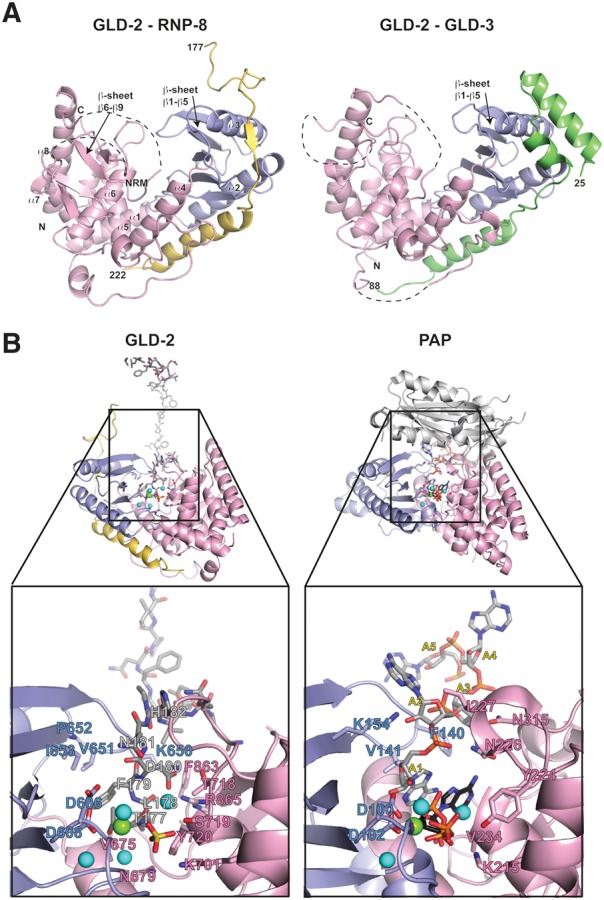
Structure of a GLD-2–RNP-8 core complex. (*A*) Ribbon diagram of the GLD-2_PAP-D_–RNP-8_GB_ complex (PDB code 5JNB, *left* panel) and GLD-2_PAP-D_–GLD-3_NT_ (PDB code 4ZRL, *right* panel) shown in the same orientation after optimal superposition of their central domains (in pink). The catalytic domains are in blue, RNP-8 in yellow, and GLD-3 in green. The N- and C-terminal residues of the proteins are labeled. Disordered loops are indicated with dashed lines. The five-stranded β-sheet (β1–β5) of the catalytic domain is indicated in both structures. In GLD-2_PAP-D_–RNP-8_GB_, an additional four-stranded β-sheet (β6–β9) is well ordered on top of the central domain. These secondary structure elements correspond to a conserved polypeptide segment between helices α7 and α8 and lay on top of helix α6. NRM, nucleotide recognition motif. (*B*) On the *left* is a zoom-in of the active site cleft of the GLD-2_PAP-D_–RNP-8_GB_ complex shown after a 180° rotation around a vertical axis with respect to the view in panel *A*. The colors are the same as in panel *A*, with the peptide of a symmetry-related RNP-8 molecule (for explanations see main text) in stick representation and with carbon atoms in gray. On the *right* is the corresponding zoom-in view from the structure of canonical PAP bound to RNA and ATP (in stick representation, with carbon atoms in gray and black, respectively) (PDB code 2Q66, Balbo and Bohm 2007). For clarity, the zoom-in view lacks the RRM domain of canonical PAP (shown as a reference in the overall view in light gray). Magnesium and water molecules are shown as green and cyan spheres, respectively. A set of important residues in both GLD-2–RNP-8_GB_ and PAP are highlighted in stick representation and labeled. Note that the place of the two most 3′ end ribonucleotides is taken by RNP-8 Phe179 and His182 (instead of the nucleotide bases) and Asn181 (instead of the nucleotide ribose moieties). The place of ATP is taken by RNP-8 Thr177, Leu178, and Asp180 (instead of the adenosine) and by Asp180 and a sulfate ion from the crystallization buffer (instead of the phosphates). Remarkably, the GLD-2_PAP-D_–RNP-8_GB_ structure also shows a similar arrangement of magnesium and water molecules in the active site as observed in the PAP-RNA-ATP structure (Balbo and Bohm 2007).

We determined the structure of GLD-2_PAP-D_–RNP-8_GB_ at a resolution of 2.5 Å and *R*_free_ of 24.2% (Table 1). The four independent copies of the complex in the asymmetric unit are very similar (with the exception that only two have the active site occupied by a magnesium ion and a peptide originating from a crystal-packing interaction, Supplemental Fig. 1D). In the text, we describe one of the complexes (chains A and E) unless otherwise specified. The final model includes residues 544–922 of GLD-2 (with the exception of missing or disordered residues between 815–860 and 879–882) and residues 177–222 of RNP-8. The overall structure of GLD-2_PAP-D_ has the typical features of nucleotidyl-transferases (for review, see Martin and Keller 2007). Briefly, the catalytic and central domains face each other forming a V-shaped cleft in between (Fig. 2A). Based on the substrate-bound structure of canonical PAP (Balbo and Bohm 2007) the cleft in GLD-2 is expected to contain the binding sites for RNA and ATP as well as the catalytic residues.

**TABLE 1.**
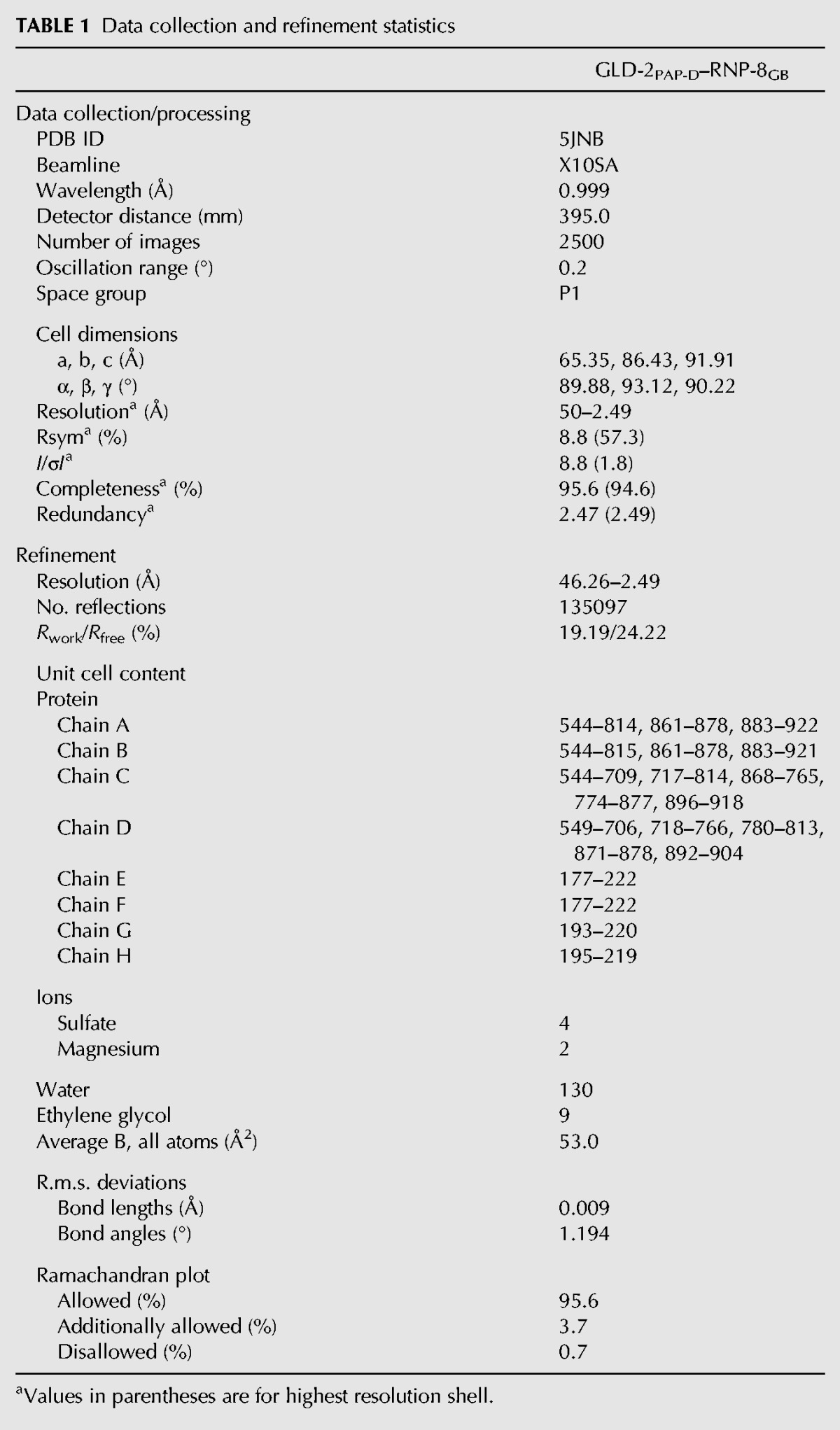
Data collection and refinement statistics

There are two major differences of GLD-2 in the RNP-8_GB_ structure as compared to the previous GLD-3_NT_ structure (Nakel et al. 2015). First, the catalytic domain is rotated toward the central domain and has a closer conformation of the active-site cleft (Fig. 2A). Second, the top of the central domain forms more rigid secondary structure elements, with a well-defined four-stranded β-sheet (β6–β9) lining the entrance of the cleft (Fig. 2A). In other nucleotidyl-transferases, this part of the molecule contains elements that determine the nucleotide specificity (the so-called nucleotide recognition motif or NRM [Schmidt and Norbury 2010]). Both differences are likely related to a crystallization effect: The active-site cleft binds the N-terminal residues of the RNP-8_GB_ polypeptide of a neighboring molecule in the lattice. With hindsight, residues 177–182 of RNP-8_GB_ mimic binding of RNA and ATP and appear essential for crystallization. This substrate mimic is remarkable (Fig. 2B), suggesting that GLD-2 was serendipitously crystallized in an active-like conformation.

**FIGURE 3.**
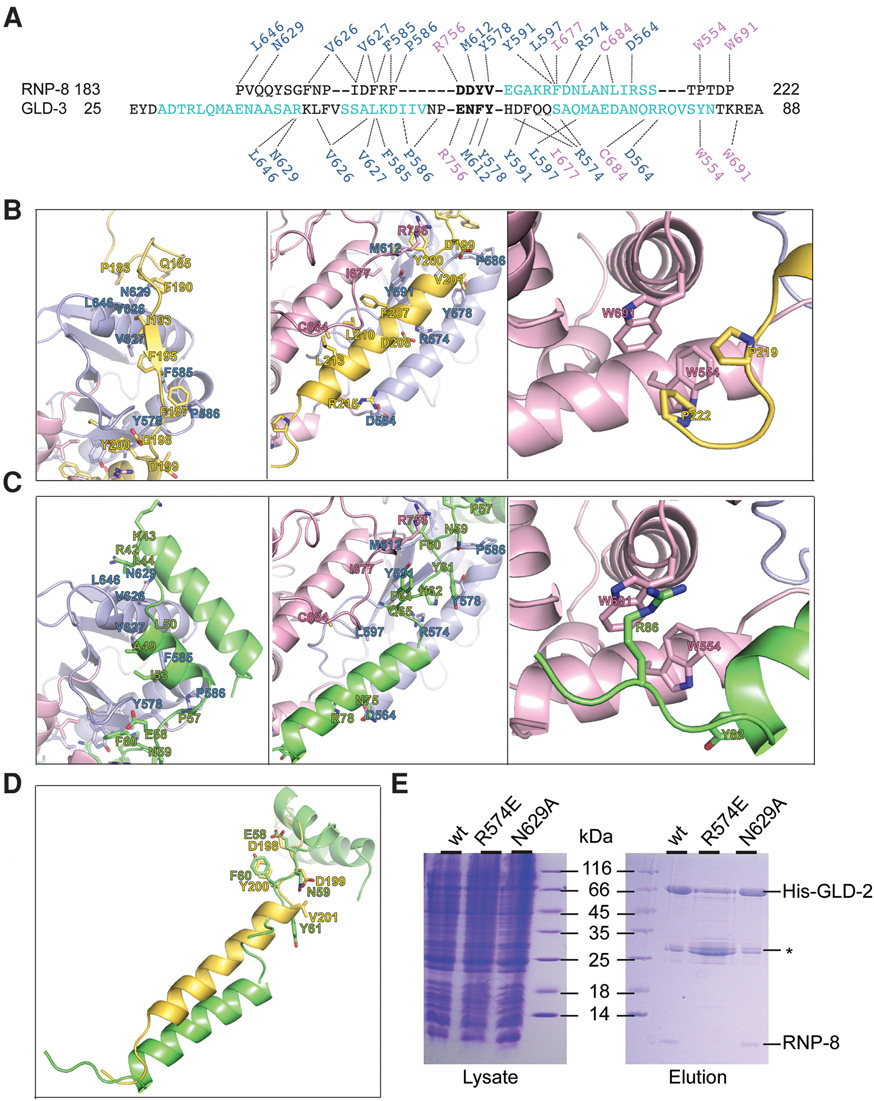
Structural basis of mutually exclusive GLD-2–RNP-8 and GLD-2–GLD-3 complex formation. (*A*) Structural alignment from *C. elegans* RNP-8_GB_ and GLD-3_NT_, with α-helical residues in cyan. Note that only the DDYV segment of RNP-8 and the ENFY segment of GLD-3 (both shown in bold letters) superpose well, with a C-α root mean square deviation of <0.1 Å. The dotted lines indicate the interactions of different GLD-3 and RNP-8 residues with the same side chains in the GLD-2 catalytic and central domains (in blue and pink *above* and *below* the sequences). (*B*) Zoom-in of RNP-8_GB_ interactions with patch 1, patch 2, and patch 3 surfaces of GLD-2 (*right*, *middle*, and *left* panels, respectively). In the *middle* panel, note that the RNP-8_GB_ helix is close to helix α4 of the GLD-2 central domain and that the GLD-2 loop containing Arg756 binds on top of the RNP-8_GB_ helix. (*C*) Corresponding zoom-in of GLD-3_NT_ interactions with the same surface patches of GLD-2 (PDB code 4ZRL, Nakel et al. 2015). In the *left* panel, two GLD-3 residues previously shown to contribute to RNA binding are indicated (Arg42, Lys43, Nakel et al. 2015). In the *middle* panel, note that the GLD-3_NT_ C-terminal helix is further apart from helix α4 of the GLD-2 central domain and that the GLD-2 loop containing Arg756 binds in between. (*D*) Superposition of RNP-8 and GLD-3 from the corresponding GLD-2–bound structures. The residues in the DDYV and ENFY segments are indicated. (*E*) Coomassie-stained 17% SDS/PAGE of His-pull-down experiments of coexpressed wild-type (wt) and mutant His-GLD-2_PAP_ with RNP-8_GB_. Total lysate control is shown on the *left*; pulled-down protein eluate is shown on the *right*. The experiment includes the disrupting GLD-2 R547E mutant (which targets a central residue at the interaction interface) and as a control the GLD-2 N629A mutant (that still supports RNP-8 binding). (*) GST-6xHis-Tag contamination.

RNP-8_GB_ wraps around GLD-2_PAP-D_, burying more than 1800 Å^2^ (35%) of its accessible surface area as calculated with PISA (Krissinel and Henrick 2007), with extensive interactions distributed over three distinct patches (Figs. 2A, 3A,B). In the N-terminal half of RNP-8_GB_, residues 183–198 adopt an extended conformation as they stretch around the helical side of the catalytic domain (patch 1). RNP-8 Pro183, Phe190, Ile193, Phe195, and Phe197 interact with exposed hydrophobic residues of GLD-2 (Fig. 3B, left panel). RNP-8_GB_ then continues with a five-turn helix that wedges in between the catalytic and central domain (patch 2, Fig. 3B, middle panel). On one side of the helix, RNP-8 Phe207, Leu210, and Leu213 are in van der Waals contacts with hydrophobic residues of GLD-2 helix α4. On the other side of the helix, RNP-8 Asp208 and Arg215 form salt bridges with residues on GLD-2 helix α2. RNP-8_GB_ ends with a short extended segment interacting with the helical domain (patch 3). Here, two proline residues (RNP-8 219 and 222) pack against two tryptophan residues of GLD-2 (Fig. 3B, right panel).

RNP-8_GB_ and GLD-3_NT_ bind with the same direction and on the same surfaces of GLD-2 (Figs. 2A, 3A–C). This is remarkable because they share little similarity in secondary structure elements. Structural comparisons show that the only portion of RNP-8_GB_ and GLD-3_NT_ that can be superimposed is a four-residue segment (centered at RNP-8_GB_ Tyr200) that binds between patch 1 and patch 2 (Fig. 3D). While both proteins form a helix upon binding to patch 2, their position and interacting residues differ (Fig. 3B,C, middle panels). Conversely, the RNP-8_GB_ and GLD-3_NT_ segments that bind to patch 1 and patch 3 have different structures but position side chains with similar chemical properties to interact with the same residues of GLD-2 (Fig. 3B,C, left and right panels). Consistent with the structural analysis, the Arg574Glu GLD-2 mutant loses the capacity of interacting with either GLD-3_NT_ (Nakel et al. 2015) or RNP-8_GB_ (Fig. 3E), whereas the Asn629Ala mutation has no influence on the binding of the co-factors (Fig. 3E; Nakel et al. 2015). Finally, most of the *C. elegans* GLD-2 residues that interact with both RNP-8 and GLD-3 are evolutionarily conserved or share similar chemical properties (Supplemental Fig. 2), raising the possibility that GLD-2 orthologs might use the same surfaces to bind other proteins. Indeed, bioinformatics analyses suggest the presence of similar hydrophobic hotspots in the GLD-2–binding region of Musashi, a GLD-2 binding partner in *Xenopus* oocytes (Supplemental Fig. 2B; Cragle and MacNicol 2014). The structural information we obtained on the *C. elegans* GLD-2 complexes may thus be useful in the future to identify and characterize other GLD-2 binding proteins in different model organisms.

The finding that RNP-8 and GLD-3 use different amino-acid sequences to compete for the same residues of GLD-2 rationalizes how they can form mutually exclusive poly(A)-polymerase complexes even if they share no apparent conservation at the sequence level. With respect to the observed genetic antagonism between GLD-3 and RNP-8 in early gametogenesis to promote opposite gamete sex (Kim et al. 2009), we presume that at the molecular level selective complex formation may be strongly influenced through developmentally induced changes in protein stoichiometry. Consistent with an abundant protein expression of all three proteins in mid-oogenesis (Eckmann et al. 2002; Wang et al. 2002; Kim et al. 2009), both complexes coexist to synergize in promoting the final stages of oogenesis (Kim et al. 2009, 2010). While a dynamic exchange between both GLD-2-interacting proteins is possible and likely, RNP-8 expression is lost during oocyte activation (Kim et al. 2010), leaving freed GLD-2 to form either further complexes with GLD-3 isoforms or other, hitherto undiscovered maternal proteins to regulate embryonic mRNA fates. In either case, RNP-8 and GLD-3 belong to distinct protein families and their additional potential interaction space is likely to funnel GLD-2_PAP_ activity to select RNA targets.

In summary, both RNP-8_GB_ and GLD-3_NT_ activate GLD-2_PAP_ with an indirect mechanism, as they both exert a stabilizing effect by covering unfavorable hydrophobic patches exposed on the surface of the poly(A)-polymerase. Only GLD-3_NT_, however, appears to stimulate GLD-2 activity directly by contributing RNA-binding residues (Nakel et al. 2015). RNP-8_GB_ lacks analogous residues and indeed has a weaker effect on GLD-2 activation in vitro. Finally, both GLD-2 complexes have a preference for RNA substrates with at least an adenosine at the 3′ end. The 3′ end substrate preference is thus a general attribute of GLD-2 that is independent of its binding partners.

## MATERIALS AND METHODS

### Protein expression and purification

The expression vector for the poly(A)-polymerase region of *C. elegans* GLD-2 was previously described (Nakel et al. 2015). It contains the GLD-2 catalytic and central domains (residues 528–923) and lacks a large flexible loop (residues 815–856), and also includes an N-terminal His-tag cleavable by tobacco etch virus (TEV) protease (pETM30 vector). The synthetic gene for *C. elegans* RNP-8 residues 171–250 was obtained from AdB Serotec/Slonomics and was subcloned in an expression vector without tag (pET21 vector). The GLD-2 and RNP-8 vectors were co-expressed in *E. coli* BL21 Gold pLyS cells (Stratagene) using Terrific Broth (TB) medium and overnight induction at 18°C. All proteins were purified with a similar protocol. Cells were lysed in a buffer containing 500 mM NaCl and protease inhibitors (Roche) and the lysate was loaded on a Ni-NTA affinity column (His60, GE Healthcare). After elution and tag cleavage, the complex was further purified by ion exchange chromatography (Q Sepharose, GE Healthcare) and size exclusion chromatography (Superdex, GE Healthcare). The complex was concentrated to 10 mg/mL in 20 mM Tris pH 7.5, 150 mM NaCl, and 4 mM DTT. GLD-2_PAP_ mutants and GLD-3_NT_ were purified as previously described (Nakel et al. 2015).

### Crystallization and structure determination

In the initial crystallization trials, GLD-2_PAP-D_–RNP-8_GB_ (10 mg/mL) were mixed with an 8mer poly(A) RNA substrate, ATP and MgSO_4_ in a 1:1,2, 1:2, and 1:4 molar ratio, respectively. Crystals of GLD-2_PAP-D_–RNP-8_GB_ grew at 20°C in a sitting-drop vapor diffusion setup using as reservoir solution 18% (v/v) polyethyleneglycol monomethylether (PEG MME) 550, 50 mM potassium nitrate, 60 mM magnesium nitrate, and 30 mM Hepes pH 7.0. Single crystals appeared in a few days. They were transferred to a cryo-protectant solution containing 15% (v/v) ethylene glycol and flash-cooled in liquid nitrogen. Native data were collected on the beamline PXII at the Swiss Light Source (Switzerland) at 100K and processed with XDS (Kabsch 2010). The crystals belong to a triclinic space-group with four molecules in the asymmetric unit. The initial phases were obtained by molecular replacement using Phaser (PHENIX) with the GLD-2_PAP_ structure (PDB ID 4ZRL) as search model (McCoy et al. 2007; Adams et al. 2010). Manual model building and iterative refinement cycles were performed using COOT (Emsley and Cowtan 2004; Emsley et al. 2010), and PHENIX (Adams et al. 2010). No RNA could be detected in the electron density. Detailed data collection and refinement statistics are summarized in Table 1.

### Biochemical and biophysical assays

Coexpressions were performed with wild-type and mutant His-GLD-2_PAP_ and RNP-8_GB_ in 200 mL TB overnight at 18°C. For pull-down assays, cells were lysed in 10 mL of 50 mM Tris pH 7.5, 500 mM NaCl, 20 mM Imidazole, and 5 mM β-Mercaptoethanol. The lysates were loaded on 700 µL of Ni-NTA resin at 4°C and the resin was washed with 10 column volumes of lysis buffer. The proteins were eluted in 2 mL of 20 mM Tris pH 7.5, 50 mM NaCl, 250 mM Imidazole, and 5 mM β-Mercaptoethanol.

Polyadenylation assays were performed in a 10-µL reaction volume containing 25 mM Tris pH 8.0, 20 mM KCl, 5 mM MgCl_2_, 10% (vol/vol) glycerol, 0.02% (vol/vol) Nonidet P-40, 1 mM DTT, and 0.05 mg/mL BSA. Final concentrations were either 20, 100, or 500 nM for proteins and 0.5 mM for ATP. The appropriate 5′-^32^P-labeled RNA synthetic oligos (from biomers.net) were added to a final concentration of 100 nM to start the reaction. Reaction mixtures were incubated at 30°C for 10 min and quenched by adding 10 µL of a buffer containing 50 mM EDTA, 0.1% SDS, and 2 mg/mL Proteinase K (New England Biolabs). Samples were incubated for 10 min at 37°C before diluting 1:3 in 95% (vol/vol) formamide, 10 mM EDTA, 0.1% bromophenol blue, 0.1% xylene cyanole, and loading 2 µL of each reaction on a 10% (wt/vol) polyacrylamide/7M urea gel. Gels were exposed overnight at −80°C to Fuji image plates and visualized using a Typhoon FLA 7000 phosphorimager (GE Healthcare).

For Thermofluor measurements, solutions containing 5 µL of protein (2 mg/mL) and 45 µL of buffer (20 mM Tris pH 7.5, 150 mM NaCl, and 10% Glycerol) with 3.9X of Sypro Orange (Invitrogen) were added to the wells of a 96-well Twin-tec plate (Eppendorf). The plate was sealed and heated in a real-time PCR system (Eppendorf) from 20°C to 80°C in increments of 0.5°C. Fluorescence changes were monitored simultaneously. The wavelengths for excitation and emission were 470 and 550 nm, respectively. A Boltzmann model was used to fit the fluorescence data after normalization and obtain the temperature midpoint for the protein unfolding transition (*T*_m_).

## SUPPLEMENTAL MATERIAL

Supplemental material is available for this article.

## DATA DEPOSITION

The coordinates and the structure factors have been deposited in the Protein Data Bank with the accession code 5JNB.

## Supplementary Material

Supplemental Material

## References

[NAKELRNA056598C1] Adams PD, Afonine PV, Bunkoczi G, Chen VB, Davis IW, Echols N, Headd JJ, Hung LW, Kapral GJ, Grosse-Kunstleve RW, 2010 PHENIX: a comprehensive Python-based system for macromolecular structure solution. Acta Crystallogr D Biol Crystallogr 66: 213–221. 20124702 10.1107/S0907444909052925 PMC2815670

[NAKELRNA056598C3] Balbo PB, Bohm A. 2007 Mechanism of poly(A) polymerase: structure of the enzyme-MgATP-RNA ternary complex and kinetic analysis. Structure 15: 1117–1131. 17850751 10.1016/j.str.2007.07.010 PMC2032019

[NAKELRNA056598C4] Barnard DC, Ryan K, Manley JL, Richter JD. 2004 Symplekin and xGLD-2 are required for CPEB-mediated cytoplasmic polyadenylation. Cell 119: 641–651. 15550246 10.1016/j.cell.2004.10.029

[NAKELRNA056598C5] Benoit P, Papin C, Kwak JE, Wickens M, Simonelig M. 2008 PAP- and GLD-2-type poly(A) polymerases are required sequentially in cytoplasmic polyadenylation and oogenesis in *Drosophila*. Development 135: 1969–1979. 18434412 10.1242/dev.021444 PMC9154023

[NAKELRNA056598C6] Cragle C, MacNicol AM. 2014 Musashi protein-directed translational activation of target mRNAs is mediated by the poly(A) polymerase, germ line development defective-2. J Biol Chem 289: 14239–14251. 24644291 10.1074/jbc.M114.548271 PMC4022889

[NAKELRNA056598C7] Crittenden SL, Eckmann CR, Wang L, Bernstein DS, Wickens M, Kimble J. 2003 Regulation of the mitosis/meiosis decision in the *Caenorhabditis elegans* germline. Philos Trans R Soc Lond B Biol Sci 358: 1359–1362. 14511482 10.1098/rstb.2003.1333 PMC1693240

[NAKELRNA056598C8] Cui J, Sartain CV, Pleiss JA, Wolfner MF. 2013 Cytoplasmic polyadenylation is a major mRNA regulator during oogenesis and egg activation in *Drosophila*. Dev Biol 383: 121–131. 23978535 10.1016/j.ydbio.2013.08.013 PMC3821703

[NAKELRNA056598C9] Eckmann CR, Kraemer B, Wickens M, Kimble J. 2002 GLD-3, a bicaudal-C homolog that inhibits FBF to control germline sex determination in *C. elegans*. Dev Cell 3: 697–710. 12431376 10.1016/s1534-5807(02)00322-2

[NAKELRNA056598C10] Eckmann CR, Crittenden SL, Suh N, Kimble J. 2004 GLD-3 and control of the mitosis/meiosis decision in the germline of *Caenorhabditis elegans*. Genetics 168: 147–160. 15454534 10.1534/genetics.104.029264 PMC1448115

[NAKELRNA056598C11] Eckmann CR, Rammelt C, Wahle E. 2011 Control of poly(A) tail length. Wiley Interdiscip Rev RNA 2: 348–361. 21957022 10.1002/wrna.56

[NAKELRNA056598C12] Emsley P, Cowtan K. 2004 Coot: model-building tools for molecular graphics. Acta Crystallogr D Biol Crystallogr 60: 2126–2132. 15572765 10.1107/S0907444904019158

[NAKELRNA056598C13] Emsley P, Lohkamp B, Scott WG, Cowtan K. 2010 Features and development of Coot. Acta Crystallogr D Biol Crystallogr 66: 486–501. 20383002 10.1107/S0907444910007493 PMC2852313

[NAKELRNA056598C14] Hansen D, Hubbard EJA, Schedl T. 2004 Multi-pathway control of the proliferation versus meiotic development decision in the *Caenorhabditis elegans* germline. Dev Biol 268: 342–357. 15063172 10.1016/j.ydbio.2003.12.023

[NAKELRNA056598C15] Kabsch W. 2010 Xds. Acta Crystallogr D Biol Crystallogr 66: 125–132. 20124692 10.1107/S0907444909047337 PMC2815665

[NAKELRNA056598C16] Kadyk LC, Kimble J. 1998 Genetic regulation of entry into meiosis in *Caenorhabditis elegans*. Development 125: 1803–1813. 9550713 10.1242/dev.125.10.1803

[NAKELRNA056598C17] Kim KW, Nykamp K, Suh N, Bachorik JL, Wang L, Kimble J. 2009 Antagonism between GLD-2 binding partners controls gamete sex. Dev Cell 16: 723–733. 19460348 10.1016/j.devcel.2009.04.002 PMC2728548

[NAKELRNA056598C18] Kim KW, Wilson TL, Kimble J. 2010 GLD-2/RNP-8 cytoplasmic poly(A) polymerase is a broad-spectrum regulator of the oogenesis program. Proc Natl Acad Sci 107: 17445–17450. 20855596 10.1073/pnas.1012611107 PMC2951458

[NAKELRNA056598C19] Krissinel E, Henrick K. 2007 Inference of macromolecular assemblies from crystalline state. J Mol Biol 372: 774–797. 17681537 10.1016/j.jmb.2007.05.022

[NAKELRNA056598C20] Kwak JE, Wang L, Ballantyne S, Kimble J, Wickens M. 2004 Mammalian GLD-2 homologs are poly(A) polymerases. Proc Natl Acad Sci 101: 4407–4412. 15070731 10.1073/pnas.0400779101 PMC384760

[NAKELRNA056598C21] Kwak JE, Drier E, Barbee SA, Ramaswami M, Yin JC, Wickens M. 2008 GLD2 poly(A) polymerase is required for long-term memory. Proc Natl Acad Sci 105: 14644–14649. 18780789 10.1073/pnas.0803185105 PMC2567210

[NAKELRNA056598C22] Martin G, Keller W. 2007 RNA-specific ribonucleotidyl transferases. RNA 13: 1834–1849. 17872511 10.1261/rna.652807 PMC2040100

[NAKELRNA056598C23] McCoy AJ, Grosse-Kunstleve RW, Adams PD, Winn MD, Storoni LC, Read RJ. 2007 Phaser crystallographic software. J Appl Crystallogr 40: 658–674. 19461840 10.1107/S0021889807021206 PMC2483472

[NAKELRNA056598C24] Moore MJ, Proudfoot NJ. 2009 Pre-mRNA processing reaches back to transcription and ahead to translation. Cell 136: 688–700. 19239889 10.1016/j.cell.2009.02.001

[NAKELRNA056598C25] Nakel K, Hartung SA, Bonneau F, Eckmann CR, Conti E. 2010 Four KH domains of the *C. elegans* Bicaudal-C ortholog GLD-3 form a globular structural platform. RNA 16: 2058–2067. 20823118 10.1261/rna.2315010 PMC2957046

[NAKELRNA056598C26] Nakel K, Bonneau F, Eckmann CR, Conti E. 2015 Structural basis for the activation of the *C. elegans* noncanonical cytoplasmic poly(A)-polymerase GLD-2 by GLD-3. Proc Natl Acad Sci 112: 8614–8619. 26124149 10.1073/pnas.1504648112 PMC4507228

[NAKELRNA056598C27] Norbury CJ. 2013 Cytoplasmic RNA: a case of the tail wagging the dog. Nat Rev Mol Cell Biol 14: 643–653. 23989958 10.1038/nrm3645

[NAKELRNA056598C28] Nousch M, Yeroslaviz A, Habermann B, Eckmann CR. 2014 The cytoplasmic poly(A) polymerases GLD-2 and GLD-4 promote general gene expression via distinct mechanisms. Nucleic Acids Res 42: 11622–11633. 25217583 10.1093/nar/gku838 PMC4191412

[NAKELRNA056598C29] Papin C, Rouget C, Mandart E. 2008 *Xenopus* Rbm9 is a novel interactor of XGld2 in the cytoplasmic polyadenylation complex. FEBS J 275: 490–503. 18177378 10.1111/j.1742-4658.2007.06216.x

[NAKELRNA056598C30] Rouhana L, Wickens M. 2007 Autoregulation of GLD-2 cytoplasmic poly(A) polymerase. RNA 13: 188–199. 17164476 10.1261/rna.333507 PMC1781367

[NAKELRNA056598C31] Sartain CV, Cui J, Meisel RP, Wolfner MF. 2011 The poly(A) polymerase GLD2 is required for spermatogenesis in *Drosophila melanogaster*. Development 138: 1619–1629. 21427144 10.1242/dev.059618 PMC3062429

[NAKELRNA056598C32] Schmidt MJ, Norbury CJ. 2010 Polyadenylation and beyond: emerging roles for noncanonical poly(A) polymerases. Wiley Interdiscip Rev RNA 1: 142–151. 21956911 10.1002/wrna.16

[NAKELRNA056598C33] Suh N, Jedamzik B, Eckmann CR, Wickens M, Kimble J. 2006 The GLD-2 poly(A) polymerase activates gld-1 mRNA in the *Caenorhabditis elegans* germ line. Proc Natl Acad Sci 103: 15108–15112. 17012378 10.1073/pnas.0607050103 PMC1622784

[NAKELRNA056598C34] Udagawa T, Swanger SA, Takeuchi K, Kim JH, Nalavadi V, Shin J, Lorenz LJ, Zukin RS, Bassell GJ, Richter JD. 2012 Bidirectional control of mRNA translation and synaptic plasticity by the cytoplasmic polyadenylation complex. Mol Cell 47: 253–266. 22727665 10.1016/j.molcel.2012.05.016 PMC3408552

[NAKELRNA056598C35] Wang L, Eckmann CR, Kadyk LC, Wickens M, Kimble J. 2002 A regulatory cytoplasmic poly(A) polymerase in *Caenorhabditis elegans*. Nature 419: 312–316. 12239571 10.1038/nature01039

[NAKELRNA056598C36] Weill L, Belloc E, Bava FA, Méndez R. 2012 Translational control by changes in poly(A) tail length: recycling mRNAs. Nat Struct Mol Biol 19: 577–585. 22664985 10.1038/nsmb.2311

