# Supplementary material for: Structural basis for the antagonistic roles of RNP-8 and GLD-3 in GLD-2 poly(A)-polymerase activity
Source: RNA. 2016 Aug;22(8):1139–45. doi: 10.1261/rna.056598.116 (PMC4931106; doi:10.1261/rna.056598.116)
Supplement: Supplemental Material [file supp_22_8_1139__index.html]

Structural basis for the antagonistic roles of RNP-8 and GLD-3 in GLD-2 poly(A)-polymerase activity — Supplemental Material 

# Structural basis for the antagonistic roles of RNP-8 and GLD-3 in GLD-2 poly(A)-polymerase activity

## Supplemental Material

**Files in this Data Supplement:**

- Supp Material.docx
