# Supplementary material for: Structural basis for the antagonistic roles of RNP-8 and GLD-3 in GLD-2 poly(A)-polymerase activity
Source: RNA. 2016 Aug;22(8):1139–45. doi: 10.1261/rna.056598.116 (PMC4931106; doi:10.1261/rna.056598.116)
Supplement: Supplemental Material [file supp_056598.116_Supplemental_Material.docx]

**Supplemental Materials**

**Structural basis for the antagonistic roles of RNP-8 and GLD-3**

**in GLD-2 poly(A)-polymerase activity**

Katharina Nakel^1^, Fabien Bonneau^1^, Claire Basquin^1^, Bianca Habermann^1^, Christian R. Eckmann^2^ and Elena Conti^1*^

^1^Max-Planck-Institute of Biochemistry, Department of Structural Cell Biology, Am Klopferspitz 18, D-82152 Martinsried, Germany

^2^Martin-Luther-University of Halle-Wittenberg, Institute of Biology, Department of Genetics, Weinbergweg 22, 06120 Halle (Saale)

* Correspondence should be addressed to Elena Conti,

[conti@biochem.mpg.de](mailto:conti@biochem.mpg.de), tel number +49 89 85783602

**Short title:** Structure of the GLD-2 – RNP-8 complex

**Keywords:** translational regulation, cytoplasmic polyadenylation, nucleotidyl-transferase, germline development, *C. elegans*

**Supplemental Figure 1**


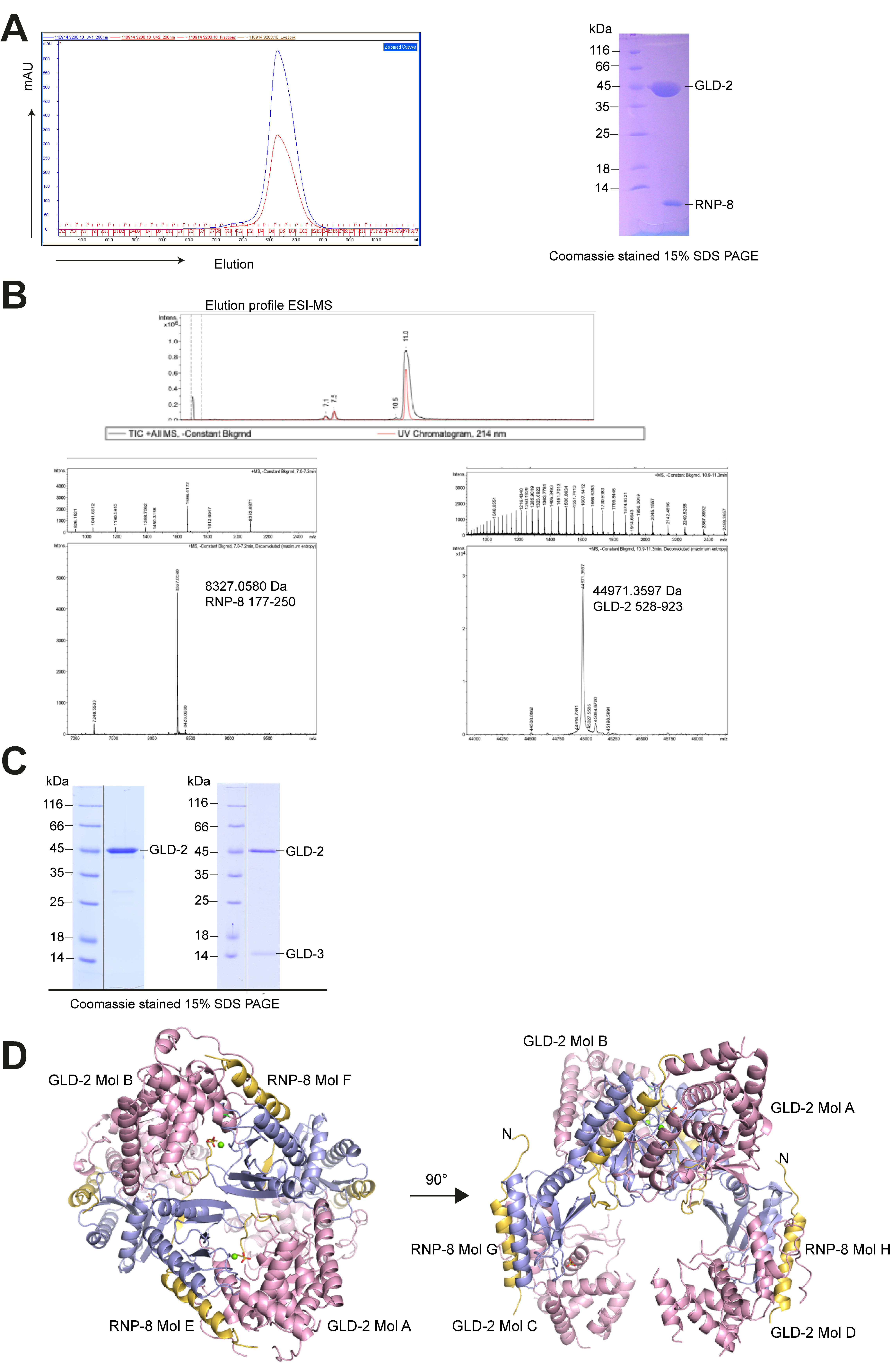


**Biochemical characterization of a minimal GLD-2 – RNP-8 complex**

1. Gel filtration profile of GLD-2_PAP_ – RNP-8_GB_ and Coomassie-stained 15% SDS-PAGE of the peak fraction.
2. Electrospray mass spectrometry data of GLD-2_PAP_ – RNP-8_GB._ Shown is the elution profile (upper panel), and the electrospray mass spectrometry data with the respective deconvoluted and reconstructed molecular mass profiles for RNP-8_GB_ (residues 177-250) and GLD-2_PAP_ (residues 528-923).
3. Coomassie-stained 15% SDS-PAGE of GLD-2_PAP_ and GLD-2_PAP_ – GLD-3_NT_ proteins used in the assays in Figure 1.
4. Ribbon diagram of the GLD-2_PAP_ – RNP-8_GB_ assembly in the crystal unit cell (PDB code 5JNB). GLD-2_PAP_ catalytic domains are shown in blue, central domains in pink, RNP-8_GB_ is shown in yellow. N-termini of RNP-8_GB_ mentioned in the text are highlighted. The picture on the right is rotated 90**°** around the horizontal axis to the picture on the left. Note that one sulfate ion (in stick representation) is present in the active site of all four GLD-2_PAP_ molecules, whereas a magnesium ion (shown as green sphere) is only present in the active site of GLD-2_PAP_ molecules (Mol) A and B. Here, the N-terminus of the opposing RNP-8_GB_ Mol E and F bind to the active site of GLD-2_PAP_ Mol B and A, respectively. In comparison, the N-termini of RNP-8_GB_ mol G and H reach into the solvent and are not ordered.

**Supplemental Figure 2**


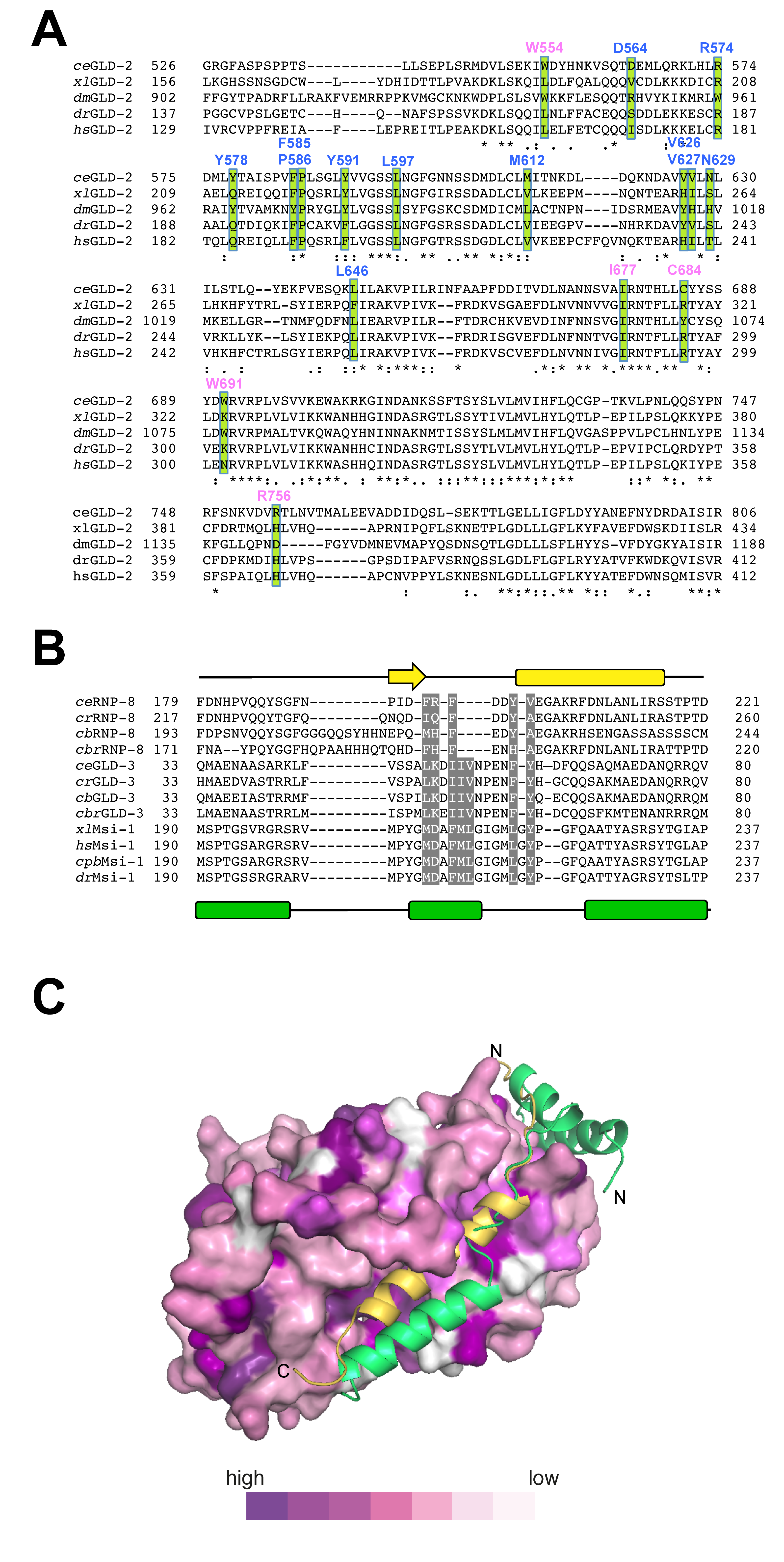


**Evolutionary conservation of GLD-2 and its binding partners**

1. Sequence alignment of the poly(A)-polymerase regions of GLD-2 orthologues from *C. elegans (ce), X. laevis (xl), D. melanogaster (dm), D. rerio (dr)* and *Homo sapiens (hs)*. Residues discussed in the text and in Figure 3 are boxed.
2. Profile-profile sequence alignment (Clustal) of nematode RNP-8 and GLD-3 with a region of vertebrate Musashi shown to interact with GLD-2 in *Xenopus* oocytes ([Cragle and MacNicol, 2014](#_ENREF_6)). *C. briggsae (cb), C. remanei (cr), C. brenneri (cbr), C. picta bellii (cpb)*
3. The structure of GLD-2_PAP_is shown in surface representation according to evolutionary conservation, from dark violet (conserved) to white (variable). The view is related to Figure 3D. The surface conservation was calculated with the ConSurf Server ([Ashkenazy et al., 2010](#_ENREF_2)) using the alignment of the five GLD-2 orthologues in Fig. S2A. For clarity, RNP-8 _GB_ and GLD-3_NT_ are shown as ribbon representations in yellow and green, respectively. Their N- and C-termini are indicated.
